# Deep 3D attention CLSTM U-Net based automated liver segmentation and volumetry for the liver transplantation in abdominal CT volumes

**DOI:** 10.1038/s41598-022-09978-0

**Published:** 2022-04-16

**Authors:** Jin Gyo Jeong, Sangtae Choi, Young Jae Kim, Won-Suk Lee, Kwang Gi Kim

**Affiliations:** 1 Department of Health Sciences and Technology, GAIHST, Gachon University, Incheon, 21999 Korea; 2 Department of Surgery and Liver Transplantation, Gil Medical Center, Gachon University, Incheon, 21565 Korea; 3 Department of Biomedical Engineering, Gil Medical Center, College of Medicine, Gachon University, Incheon, 21565 Korea; 4 Department of Surgery, Gil Medical Center, College of Medicine, Gachon University, Incheon, 21565 Korea

**Keywords:** Medical imaging, Biomedical engineering

## Abstract

In living-donor liver transplantation, the safety of the donor is critical. In addition, accurately measuring the liver volume is significant as the amount that can be resected from living donors is limited. In this paper, we propose an automated segmentation and volume estimation method for the liver in computed tomography imaging based on a deep learning-based segmentation network. Our framework was trained using the data of 191 donors, achieved a dice similarity coefficient of 0.789, 0.869, 0.955, and 0.899, respectively, in the segmentation task for the left lobe, right lobe, caudate lobe, and whole liver. Moreover, the R^2 score reached 0.980, 0.996, 0.953, and 0.996 in the volume estimation task. We demonstrate that our approach provides accurate and quantitative liver segmentation results, reducing the error in liver volume estimation. Therefore, we expected to be used as an aid in estimating liver volume from CT volume data for living-donor liver transplantation.

## Introduction

Liver transplantation is the definitive treatment that increases the survival rate of patients with end-stage liver cancer and cirrhosis^1,2^. Major hepatic resection is increasingly performed due to advances in surgical devices and preoperative diagnostic imaging that allows accurate delineation of anatomical variants and liver volume assessment in living liver transplantation^3^. Postoperative morbidity is mainly due to hepatic dysfunction and bile leaks following extended liver resection^4^. Thus, accurately estimating the remnant liver is critical to avoid liver failure as the most serious complication.

Liver volumetry is performed for the donor liver, to calculate the graft volume and remnant liver to avoid graft size mismatch^5^. The safety of the liver donor is a major concern in liver transplantation. Overestimation of the donor liver can result in excess hepatic resection, while underestimation of the recipient’s standard liver volume can result in small-for-size graft syndrome^5,6^. Therefore, to measure the liver volume accurately, it is necessary to perform an accurate liver segmentation.

In general, liver segmentation approaches include manual segmentation and automated segmentation. Manual segmentation is time-consuming, poorly reproducible, and results vary with operators, and it is a task that requires high-level technical skills. Automated segmentation may reduce errors from manual segmentation^7^.

Recently, convolutional neural networks (CNNs) have shown promise for performing various tasks in medical imaging^8,9^. Traditional machine learning and image processing, in which limited features are extracted for segmentation, require cautious design by a researcher. In contrast, CNNs have the ability to learn relevant features, which define the anatomical structure and tissue of the liver, to segment the liver automatically in a training dataset of computed tomography (CT) volumes. For instance, U-Net is an encoder‒decoder model that improves the performance by applying a skip connection to compensate for missing data due to convolution^10^. Recently, state-of-the-art approaches that apply an attention-mechanism (AM), deep supervision (DS), and nested architecture techniques, have improved segmentation performance^11–14^.

An attention U-Net’s AM approach demonstrated an increase in pancreas segmentation performance in TCIA Pancreas-CT Dataset^11^. The pancreas is a small organ, with a shape that differs across patients. An attention U-Net improved performance of segmentation of pancreas of various shapes and small sizes, by using an AM with 1 × 1 convolution layer and a sigmoid activation function to reduce background weight and to preserve foreground weight.

An Attention U-Net^++^ improved liver segmentation performance by applying an AM, DS, and nested architecture^13^. In order to integrate semantic information from feature maps at different semantic levels from output nodes, the convergence speed of the loss and the segmentation accuracy was improved by optimizing the losses obtained at each level. These approaches have the advantages of focusing on a small organ or tissue and accelerate optimal convergence^11–14^.

Recently, several studies have been conducted for inter-frame and inter-slice learning from video data and three-dimensional (3D) volumetric data using convolutional long short-term memory (CLSTM)^15–19^. LSTM is usually used in natural language processing and is mainly used for continuous data or for data with a temporal sequence. CLSTM was developed for CNN models^20–22^. A previous study proposed a 3D segmentation approach that combined CLSTM in 3D fungus datasets. The proposed approach achieved higher performance than the generalized 3D CNN. The CLSTM approach demonstrated that it could learn efficiently in the context of inter-slice and 3D volume data.

In this study, we describe a method for automatic liver segmentation from abdominal CT volume data, in comparison with human liver volumetry conducted by a single liver transplant surgeon. First, we proposed a deep 3D CNN model comprised of AM and DS to achieve fast and accurate segmentation of CT volumetric data. Second, we used CLSTM in our network to learn the inter-slice context. Third, we evaluated and compared liver segmentation accuracy and liver volumetry with human-based volumetry data. Finally, we evaluated the CNN for left, right, and caudate lobe volumetry to show its clinical significance.

## Methods

### Data sources and patient demographics

In this study, our dataset was obtained 191 abdominal CT data from liver transplant adult donors (mean age, 31 years ± 11 years; 58% male; mean weight, 65.9 ± 11.4 kg) from 2005 to 2017 at Gachon University Gil Hospital. This study was conducted in accordance with the Declaration of Helsinki and approved by the Institutional Review Board of the Gil Medical Center (IRB No. GBIRB2021-229), and written informed consent was obtained from all the participants. All images were deidentified before inclusion in this study. The demographics and other characteristics of each cohort are summarized in Table 1. The imaging system is summarized in Table 2.

**Table 1:** Characteristics of patients whose image data were used in this study.

	Females	Males	All
No. of patients	80	111	191
Age (years)	33.1 (10.7)	31.0 (12.1)	31.8 (11.5)
Body height (cm)	161.9 (5.5)	172.8 (5.8)	168.4 (7.8)
Body weight (kg)	58.9 (7.8)	70.8 (10.9)	65.9 (11.4)

Data summarized as mean (± SD).

**Table 2:** Imaging systems and parameters.

Imaging systems and parameters	Training/validation at our institution
Imaging system	Siemens
In-plane resolution (mm)	0.5‒0.9
Section thickness (mm)	5.0
Collimation (mm)	NA
Tube current (mAs)	50–550
Tube voltage (kVp)	90–120

### Datasets and data pre-processing

**Figure 1:**
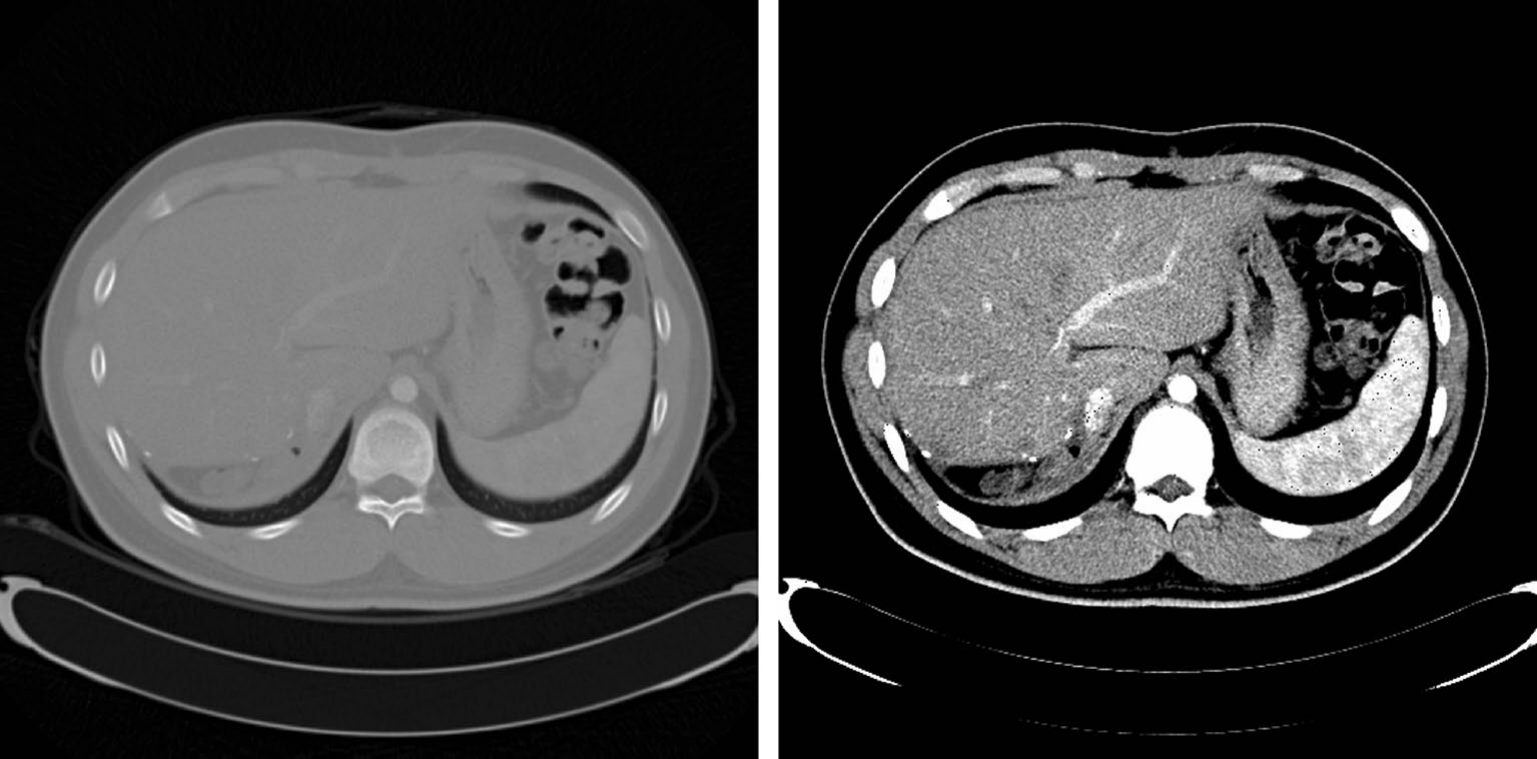
Comparison between the raw computed tomography axial view images (left) and with the window setting (right).

In this study, the window level and window width were set to clearly observe the liver on CT (window level: 50, window width: 180)^23^. Figure 1 shows the result of the window setting. Furthermore, CT data had the same 512 × 512 in-plane resolutions, but due to the computational limitation of the graphics card, the image was resized to a resolution of 256 × 256. Finally, the image and ground truth data had a shape of 64 × 256 × 256 × 1 and were divided into train: validation: test sets in the ratio of 70: 15: 15.

### Liver segmentation using DALU-Net

**Figure 2:**
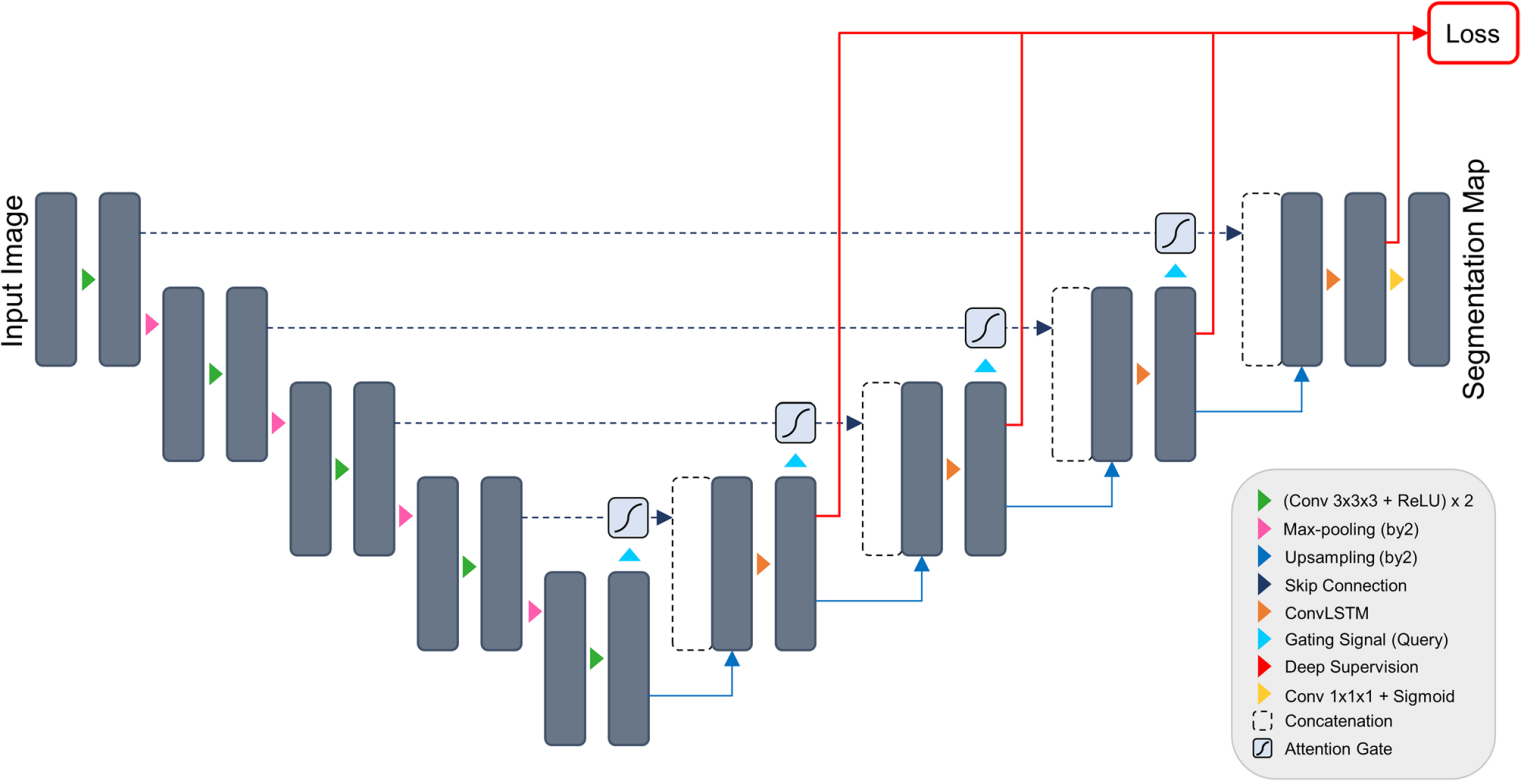
The proposed DALU-Net architecture.

The proposed model Deep Attention LSTM U-Net (DALU-Net) had an architecture similar to the standard U-Net, consisting of an encoder and a decoder^10^. The encoder could extract more complex hierarchical features and obtain contextual information. The decoder deconvolutes the features extracted by the encoder to reconstruct the size of the volume reduced by the convolution operation. In addition, it concatenates hierarchical features using the skip connection at every level of encoder and the decoder. Information about localization lost due to convolution and pooling layers in the encoder, can be corrected, and the network can segment objects more accurately. Figure 2 illustrated the DALU-Net architecture.

DALU-Net is a model that combines AM, DS, and CLSTM techniques. AM uses a module called attention gate (AG) to skip connections between the up-sampling layer and encoder. The CLSTM was used in the feature map from AG and was used only in the decoder. DS was used for fast convergence of the model, and the loss is calculated at every level of the decoder, and the final loss was calculated as the sum of each loss.

**Figure 3:**
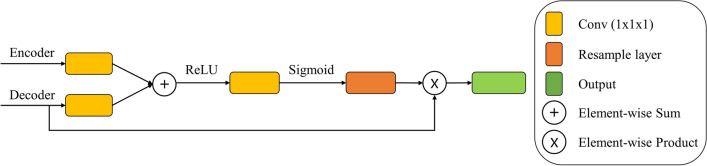
Schematic of attention mechanism.

AM is commonly used for machine translation^24^ and classification in natural language processing and graph neural networks^25,26^. Recently, AM has been variously used in semantic segmentation and classification tasks^27^. AM has demonstrated improved accuracy in the medical image^11,13,28,29^. In the image segmentation and classification task, the AM was designed to generate an attention map by analyzing the gradient of the output class score for the input image, reduce the weight of the background by multiplying it with the input image, and focus on the object. The details of the AM are shown in Fig. 3.

DS supervises the CNN's hidden layer in the segmentation task, to speed up convergence and resolve the gradient-vanishing problem^12,17,30^. The red arrow in Fig. 2, the loss is calculated using a sigmoid function for every level of the decoder, and the final loss was determined by summing each loss. It has the advantage of allowing the final loss to converge rapidly by optimizing at each level. In this study, DS was applied to the output values from the attention gate of each level.

### Liver volumetry

The segmented liver area was calculated using the proposed model on the axial image. The liver volume was calculated for each image by multiplying the liver area and the section thickness. Thus, the whole liver volume was estimated by adding the liver volume of all images.

### Ground truth for liver segmentation and volumetry

The liver was manually labeled on all CT images under the supervision of a liver transplant surgeon with more than 5 years of experience in CT analysis related to liver transplantation. ImageJ software was used for manual segmentation (NIH, Bethesda, MD, USA). For liver volumetry, the calculated liver volume, based on manual segmentation, was used as the reference standard (as described in “Liver Volumetry” section).

### Ground truth for left and right and caudate lobe

**Figure 4:**
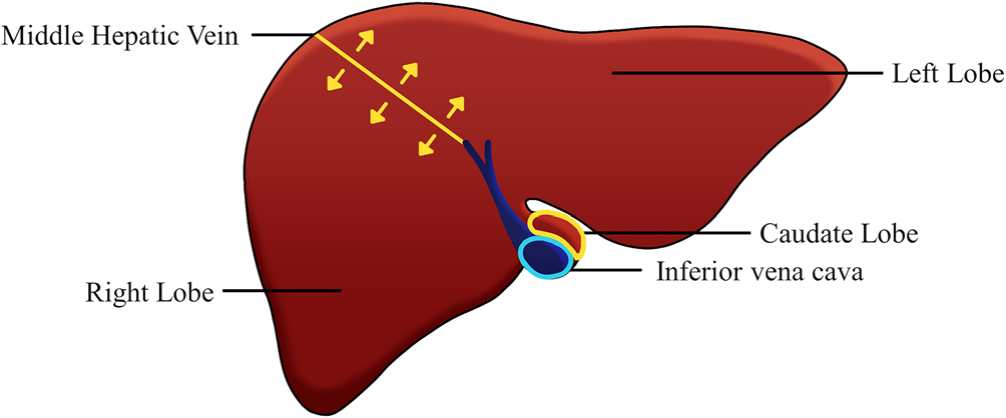
Illustration of the ground truth for the left lobe, right lobe, and caudate lobe at the axial view.

In this study, a comparative analysis was performed for segmentation and volume measurements for the left, right, and caudate lobe regions, according to the anatomical structure of the liver^31,32^. The left lobe was defined as the upper region, above the middle hepatic vein, and the lower region was defined as the right lobe. The caudate lobe was located to the left of the inferior vena cava (IVC), without overlapping the left lobe in the coronal view^31,32^. Figure 4 schematically illustrates the ground truth for the left lobe, right lobe, and caudate lobe.

### Implementation details

The DALU-Net was implemented in Python 3.7, TensorFlow 2.1.3, Keras 2.3.1, and was run on four NVIDIA Tesla V100 GPU with 5120 cores and 32 GB of memory. Our networks were trained using the Adam optimizer to minimize the dice loss^33,34^. When the loss was not minimized over 10 epochs, the learning rate was reduced by multiplying the initial learning rate by 0.1, with an initial value of 0.01. We terminated the process early when the loss did not improve for 30 epochs in the training procedure. Our network terminated the training procedure early in 90–150 epochs.

### Evaluation metrics

We evaluated the performance of the proposed approach. The evaluation metrics included the dice similarity coefficient (DSC), intersection over union (IOU), and Hausdorff distance^35^. The DSC was defined as the volume of overlap between the CNN and manual labeling segmentations divided by the average of the segmentation volume of the two methods. The IOU was defined as a mathematical indicator of how much the two objects’ positions coincide. The Hausdorff distance was defined as the difference measured between two subsets of metric space.

### Informed consent

Informed consent was obtained from all subjects involved in the study.

## Results

### Liver segmentation accuracy

**Figure 5:**
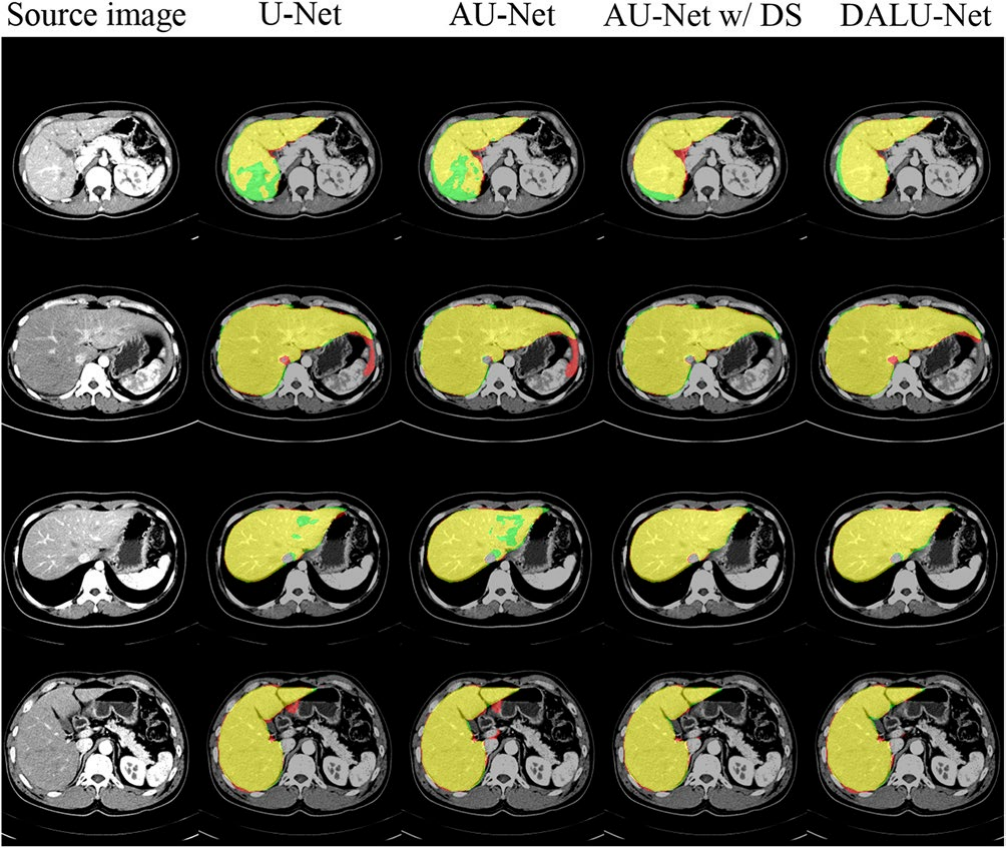
Examples of liver segmentation results at the axial view images for the various models. Segmentation results are color-coded: green is ground truth, red is automated segmentation, and yellow is the overlap between the ground truth and automated segmentation.

We analyzed the accuracy of the liver segmentation network. To this end, we compared the 3D U-Net, Attention U-Net, Attention U-Net with DS, and DALU-Net. Table 3 shows the performance of our method in the four validation datasets: (a) left lobe, (b) right lobe, (c) caudate lobe, (d) whole liver. We used evaluation metrics to measure the accuracy of the segmentation results: the higher the value of the evaluation metrics, the better the segmentation results. On the other hand, the smaller the Hausdorff distance was, the better were the results. Our method performed better than 3D U-Net in terms of segmentation accuracy (DSC), with 7% and 10% improvement in the whole liver and caudate lobe datasets, respectively. Figure 5 shows the visual assessment of the automated liver segmentation.

**Table 3:** Segmentation results of an ablation study of the proposed model on the four validation datasets.

Dataset	Model	Recall	Precision	DSC	IOU	HD (mm)
Left lobe	3D U-Net	0.838 ± 0.124	0.803 ± 0.081	0.771 ± 0.082	0.783 ± 0.127	6.895 ± 1.794
	AU-Net	0.838 ± 0.122	0.808 ± 0.086	0.785 ± 0.081	0.776 ± 0.128	6.691 ± 1.785
	AU-Net w/ DS	0.847 ± 0.121	0.812 ± 0.083	0.787 ± 0.082	0.779 ± 0.128	6.630 ± 1.732
	DALU-Net	0.851 ± 0.121	0.806 ± 0.083	0.789 ± 0.084	0.787 ± 0.128	6.629 ± 1.696
Right lobe	3D U-Net	0.966 ± 0.069	0.786 ± 0.067	0.855 ± 0.065	0.821 ± 0.138	5.144 ± 1.635
	AU-Net	0.966 ± 0.067	0.807 ± 0.072	0.867 ± 0.037	0.856 ± 0.115	5.125 ± 1.714
	AU-Net w/ DS	0.972 ± 0.063	0.804 ± 0.071	0.868 ± 0.066	0.827 ± 0.121	5.087 ± 1.731
	DALU-Net	0.975 ± 0.058	0.805 ± 0.071	0.869 ± 0.067	0.896 ± 0.141	5.074 ± 1.705
Caudate lobe	3D U-Net	0.825 ± 0.326	0.793 ± 0.316	0.849 ± 0.307	0.819 ± 0.316	2.133 ± 1.476
	AU-Net	0.905 ± 0.241	0.817 ± 0.209	0.922 ± 0.236	0.896 ± 0.236	1.557 ± 1.273
	AU-Net w/ DS	0.914 ± 0.229	0.822 ± 0.217	0.929 ± 0.215	0.907 ± 0.222	1.583 ± 1.221
	DALU-Net	0.943 ± 0.183	0.877 ± 0.174	0.955 ± 0.167	0.936 ± 0.181	1.238 ± 1.131
Whole liver	3D U-Net	0.899 ± 0.155	0.850 ± 0.287	0.822 ± 0.295	0.770 ± 0.288	3.236 ± 1.256
	AU-Net	0.891 ± 0.162	0.893 ± 0.241	0.852 ± 0.258	0.800 ± 0.259	3.280 ± 0.982
	AU-Net w/ DS	0.922 ± 0.110	0.907 ± 0.213	0.887 ± 0.218	0.840 ± 0.219	2.980 ± 0.784
	DALU-Net	0.923 ± 0.120	0.924 ± 0.188	0.899 ± 0.201	0.855 ± 0.205	2.762 ± 0.728

### Effect of layer on segmentation accuracy

To prove the effectiveness of our proposed method, we performed an ablation study on our dataset. We compared different configurations with the baseline of 3D U-Net. The result of the ablation study is summarized in Table 3. For the liver, the DSC increased from 0.822 to 0.852 with AG. Then, we evaluated the contribution of DS. By using DS, the DSC increased from 0.852 to 0.887. Finally, we analyzed the importance of CLSTM in our proposed model and showed that the DSC increased from 0.887 to 0.899.

### Liver volumetry accuracy

**Figure 6:**
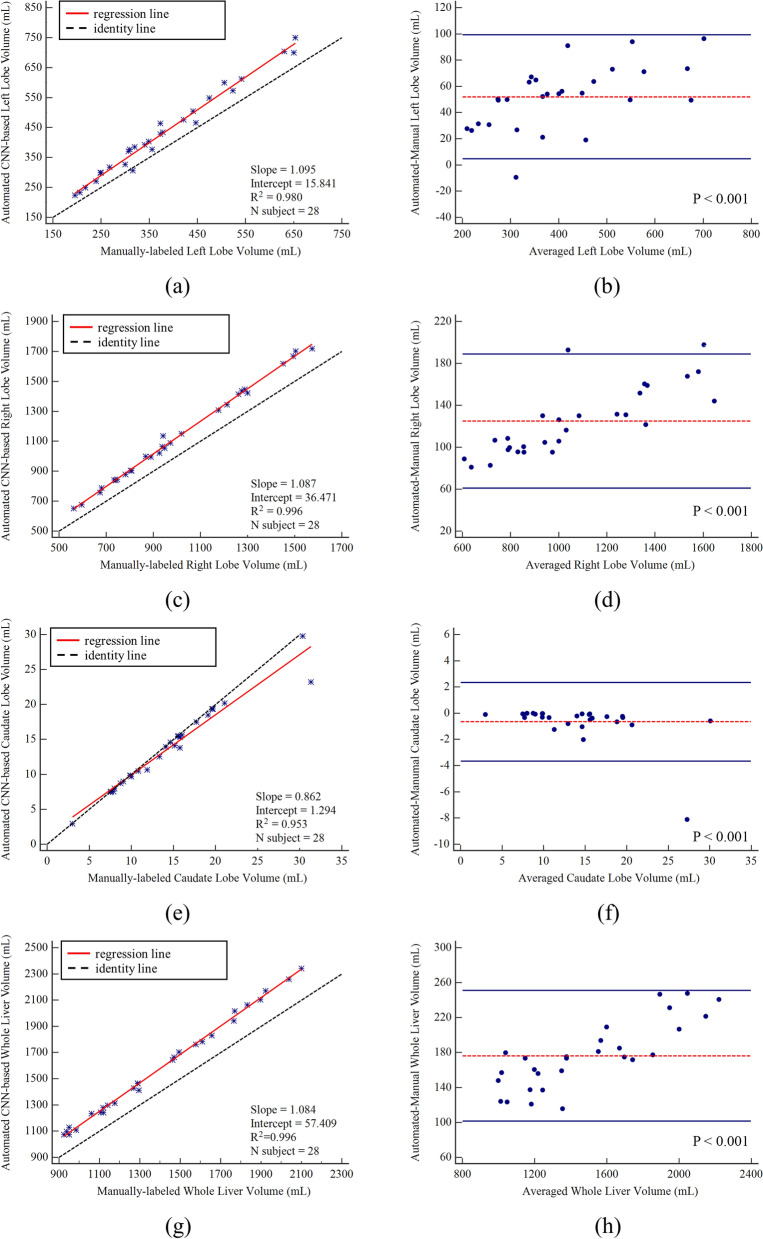
Agreement of liver volume assessments between convolutional neural network-predicted and manual liver segmentation. (**a**) Linear regression and (**b**) Bland‒Altman analysis of left lobe volume assessments. (**c**) Linear regression and (**d**) Bland‒Altman analysis of right lobe volume assessments. (**e**) Linear regression and (**f**) Bland‒Altman analysis of caudate lobe volume assessments. **(g**) Linear regression and (**h**) Bland‒Altman analysis of whole liver volume assessments.

The comparison of between human volumetry and automated liver volumetry is summarized in Fig. 6. Left lobe volumes obtained by manual segmentation ranged from 195 to 653 mL (mean volume, 379 ± 130 mL), and that obtained by automated segmentation ranged from 223 to 749 mL (mean volume, 431 ± 144 mL). Manual and automated left lobe volumetry correlated strongly (R^2 = 0.980, slope = 1.095, intercept = 15.841, Fig. 6a). When compared with the manual method, the automated method resulted in a slight underestimation of the left lobe volume (bias = 51.9 mL, *P* < 0.001), and the 95% limits of agreement (LoA) were 4.5 mL and 99.3 mL (Fig. 6b).

Right lobe volumes obtained by manual segmentation ranged from 562 to 1573 mL (mean volume, 1006 ± 290 mL), and that obtained by automated segmentation ranged from 651 to 1717 mL (mean volume, 1130 ± 316 mL). Manual and automated right lobe volumetry correlated strongly (R^2 = 0996, slope = 1.087, intercept = 36.471, Fig. 6c). When compared with the manual method, the automated method resulted in a slight underestimation of right lobe volume (bias = 124.8 mL, *P* < 0.001), and the 95% LoA were 61.0 mL and 188.6 mL (Fig. 6d).

Caudate lobe volumes obtained by manual segmentation ranged from 3 to 31 mL (mean volume, 14 ± 6 mL), and that obtained by automated segmentation ranged from 3 to 30 mL (mean volume, 13 ± 5 mL). Manual and automated caudate lobe volumetry correlated strongly (R^2 = 0.953, slope = 0.862, intercept = 1.294, Fig. 6e). When compared with the manual method, the automated method resulted in a slight underestimation of the caudate lobe volume (bias = − 0.7 mL, *P* < 0.001), and the 95% LoA were − 3.7 mL and 2.3 mL (Fig. 6f).

Whole liver volumes obtained by manual segmentation ranged from 924 to 2100 mL (mean volume, 1400 ± 357 mL), and that obtained by automated segmentation ranged from 1072 to 2340 mL (mean volume, 1576 ± 388 mL). Manual and automated whole liver volumetry correlated strongly (R^2 = 0.996, slope = 1.084, intercept = 57.409, Fig. 6g). When compared with the manual method, the automated method resulted in a slight underestimation of the whole liver volume (bias = 176 mL, *P* < 0.001), and the 95% LoA were 101.3 mL and 250.8 mL (Fig. 6h).

## Discussion

In this study, we showed that our proposed DALU-Net model, based on AG, DS, and CLSTM, can be trained to perform automated and accurate liver segmentation using abdominal CT data. We achieved high performance with a segmentation accuracy DSC of 0.899 for the whole liver. Compared to the reference model, 3D U-Net, the performance of the proposed model was improved by 7%. Table 3 presents the results of the AG, DS, and CLSTM models to the basic 3D U-Net model; whole liver segmentation accuracy improved by 3%, 3%, and 1%, respectively. Figure 5 presents the training results of the model. For the proposed DALU-Net model, segmentation results were more accurate than those of other models. We demonstrated that the segmentation accuracy increased with addition of each method.

Figure 6g, h show the results of the linear regression analysis between the human-based whole liver volumetry and the volume of the automatically segmented liver. The slope of the regression curve was 1.084 and ${R}^{2}$ was 0.996, indicating that the two values were similar. However, the regression line was located above the identity line. The automated method overestimated liver volume as compared to manual liver volumetry. Since the slice thickness was relatively large (5 mm), it is assumed that there were many false positives in the liver segmentation results, due to insufficient information for a 3D context. However, results of the Bland‒Altman analysis showed that most values were within the 95% confidence interval. It can be confirmed that manual and automatic volume estimations are similar.

In previous liver segmentation studies, whole liver segmentation accuracy and volumetry were compared. In contrast, we further compared the segmentation accuracy and volumetry for the left, right, and caudate lobes. In the three-validation datasets, defined according to the anatomy of the liver, the DSC was 0.789, 0.869, and 0.955 for the left, right, and caudate lobes, respectively. There was no significant difference between the DSCs of the proposed model and those of the 3D U-Net model in the left and right lobes. In contrast, the DSC of the proposed model for the caudal lobe data was improved by 10% as compared to that of the 3D U-Net model. The caudate lobe is attached to the IVC. It is smaller than the left and right lobe, and with a 5-mm section thickness, it has a small number of slices in terms of CT volume data. Consequently, it was difficult to perform inter-slice learning with a general 3D convolution layer. The proposed model, using CLSTM, was assumed to be capable of inter-slice learning and achieved high accuracy.

Linear regression analysis in the three validation datasets yielded the slope of regression lines of 1.095, 1.087, and 0.862, which are close to 1, and ${R}^{2}$ of 0.980, 0.996, and 0.953, for the left, right, and caudate lobes, respectively. This indicated that manual and automated volumetry yielded similar results. In addition, using Bland‒Altman analysis, we demonstrated that the gap between the manual liver volume and the CNN-based liver volume is small, as most values were within the 95% confidence interval.

We have several limitations to the dataset. The contrast agent injection amount and the scan time after injection are different, so it has various contrast in the dataset^36^. In addition, there have several cases with low accuracy because the section thickness was 5 mm and there were not many slices overlapping the heart in the CT volume data, so it was not possible to distinguish between the myocardium and the liver. Additional work may be required to improve performance in these problems. More extensive testing helps to reduce the failed cases of CNN, so inform the choice of additional training data to further improve model performance.

In summary, the proposed DALU-Net, using AM, DS, and CLSTM, can perform liver segmentation and volumetry using abdominal CT volume data. Furthermore, the volume of the left, right, and caudate lobes can be measured, according to the anatomical structure of the liver. Thus, our approach can be used for estimating liver volumes from CT data to assist in planning of living-donor liver transplantation.

## Data Availability

The CT volume data used to support the findings of this study are available upon request from the corresponding author.
